# Incidence and Short-term Mortality From Perforated Peptic Ulcer in Korea: A Population-Based Study

**DOI:** 10.2188/jea.JE20120056

**Published:** 2012-11-05

**Authors:** SeungJin Bae, Ki-Nam Shim, Nayoung Kim, Jung Mook Kang, Dong-Sook Kim, Kyoung-Min Kim, Yu Kyung Cho, Sung Woo Jung

**Affiliations:** 1 Health Insurance Review and Assessment Service, Research and Development Center, Seoul, South Korea; 2 Department of Internal Medicine, Ewha Womans University School of Medicine, Seoul, South Korea; 3 Department of Internal Medicine, Seoul National University Bundang Hospital, Seongnam, Gyeonggi-do, South Korea; 4 Seoul National University Hospital Healthcare System Gangnam Center, Seoul, South Korea; 5 Department of Internal Medicine, The Catholic University of Korea, Seoul, South Korea; 6 Department of Internal Medicine, Korea University College of Medicine, Ansan, Gyeonggi-do, South Korea

**Keywords:** peptic ulcer perforation, incidence, mortality, population

## Abstract

**Background:**

Perforated peptic ulcer (PPU) is associated with serious health and economic outcomes. However, few studies have estimated the incidence and health outcomes of PPU using a nationally representative sample in Asia. We estimated age- and sex-specific incidence and short-term mortality from PPU among Koreans and investigated the risk factors for mortality associated with PPU development.

**Methods:**

A retrospective population-based study was conducted from 2006 through 2007 using the Korean National Health Insurance claims database. A diagnostic algorithm was derived and validated to identify PPU patients, and PPU incidence rates and 30-day mortality rates were determined.

**Results:**

From 2006 through 2007, the PPU incidence rate per 100 000 population was 4.4; incidence among men (7.53) was approximately 6 times that among women (1.24). Incidence significantly increased with advanced age, especially among women older than 50 years. Among 4258 PPU patients, 135 (3.15%) died within 30 days of the PPU event. The 30-day mortality rate increased with advanced age and reached almost 20% for patients older than 80 years. The 30-day mortality rate was 10% for women and 2% for men. Older age, being female, and higher comorbidity were independently associated with 30-day mortality rate among PPU patients in Korea.

**Conclusions:**

Special attention should be paid to elderly women with high comorbidity who develop PPU.

## INTRODUCTION

Perforated peptic ulcer (PPU) is a serious medical condition with a mortality rate as high as 25%.^1^^,^^2^ With the introduction of proton pump inhibitors (PPIs) and increased knowledge of PPU etiology, the incidence of PPU has reportedly decreased in Western countries.^3^^,^^4^ However, PPU incidence remains relatively high among seniors. Because of poor PPU outcomes in this age group,^1^^,^^5^ this condition is still a significant concern for clinicians and policymakers. Despite the clinical importance of PPU, only a few studies have used nationally representative samples to investigate Asian patients. Li et al reported that the results of PPU outcome studies might not be applicable to races or populations in different countries, which suggests a need for country-specific studies.^6^

Predisposing factors for PPU include age older than 60 years, smoking, use of non-steroidal anti-inflammatory drugs (NSAIDs), and chronic stress.^7^ In addition, about 83% of PPU cases are associated with *Helicobacter pylori* (*H. pylori*).^8^ PPU is a special concern for Koreans because the prevalence of *H. pylori* is 59.6% among Koreans older than 16 years.^9^ In addition, Korea is expected to experience a rapid increase in its aged population by 2026. Since elderly adults are more likely to have comorbidities such as heart disease, cerebrovascular disease, and arthritis, they are more likely to be exposed to NSAIDs and are thus vulnerable to PPU.

Because PPU is a rare medical condition, population-based databases such as the Korean National Health Insurance (NHI) claims database could be a valuable data source for population-based studies investigating the incidence of PPU and 30-day mortality rates of PPU patients. However, the internal validity of claims databases has been questioned due to inaccurate or incomplete coding.^10^ In addition, the positive predictive value (PPV) of the International Classification of Disease (ICD) codes for diagnosing PPU using other administrative database has been mediocre.^11^^,^^12^ Combining procedure codes with diagnostic codes in the identification of PPU cases has been suggested.^10^ Based on this background, the aims of this study were, first, to estimate age- and sex-specific PPU incidence rates and short-term mortality from PPU in Korea using the NHI claims database and, second, to identify risk factors for mortality associated with PPU events.

## METHODS

### Study population and data source

We conducted a retrospective population-based study using the Korean NHI claims database from January 2006 through December 2007. This database contained information from a population of 48.4 million as of 2007. Demographic characteristics, health care utilization records, and pharmacy records were provided by the NHI claims database from 2006 to 2007. The database includes but is not limited to demographic information, diagnostic codes, procedures codes, claim dates, and pharmacy claim records. The NHI claims database contains information for 97% of the total Korean population.

More than 99% of claims have been submitted electronically since 2005, and all data files (eg, pharmacy, hospital, and demographic files) could be linked by unique patient identification numbers. A mortality database, which includes date of death and personal identification number, has been merged with the NHI database, using personal identification numbers. However, personal identification numbers have been encoded and blocked to protect patient privacy. Thus, the present authors were blinded to all complete personal identification numbers. Because we were working with a claims database, details on clinical and other relevant information (eg, smoking and alcohol consumption habits) were not available. The study outcome variable was death within 30 days of a PPU event. Cause of death was not available in the database.

### Diagnostic algorithm

**Figure 1.**
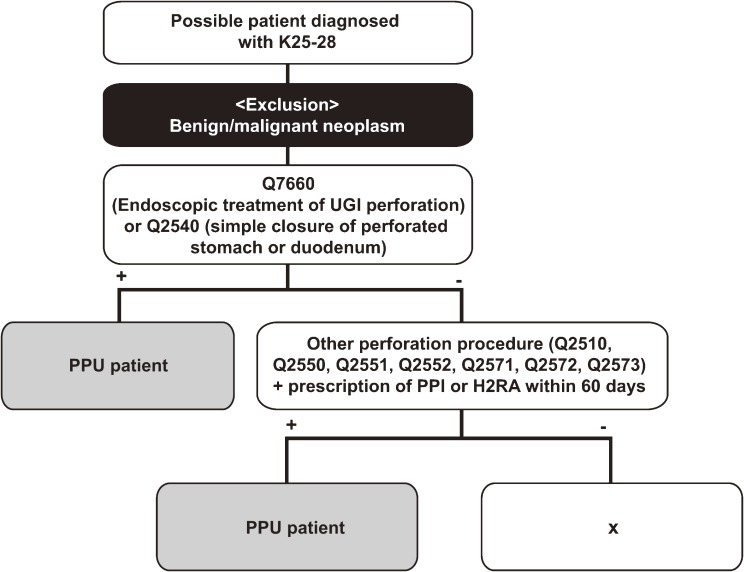
Diagnostic algorithm for patients with perforated peptic ulcer. The algorithm was developed to identify PPU patients, using data from the Korean NHI claims database. Q2510, gastrotomy; Q2550, vagotomy; Q2551, truncal vagotomy with gastrojejunostomy or pyloroplasty; Q2552, truncal vagotomy with gastrectomy; Q2571, gastroduodenostomy; Q2572, gastrojejunostomy; Q2573, gastrojejunostomy (Roux-en-Y jejunostomy). UGI, upper gastrointestinal; PPU, perforated peptic ulcer; PPI, proton pump inhibitor; H2RA, H2-receptor antagonist.

A diagnostic algorithm (Figure 1) using the NHI claims database to identify PPU patients was constructed based on the opinions of experts and guidelines for PPU clinical practice.^4^^,^^13^^,^^14^ The algorithm is the combination of diagnostic codes, procedure codes, and drug utilization patterns. That is, potential PPU candidates were patients who were hospitalized for diseases corresponding to ICD-10 codes K25.X, 26.X, 27.X, and 28.X (see eTable 1 for details) as their primary or secondary diagnosis^15^^,^^16^ and had undergone PPU-specific procedures such as endoscopic treatment of upper gastrointestinal perforation (Q7660) or simple closure of a perforated stomach or duodenum^17^ (Q2540). Individuals were defined as PPU patients if they had undergone specific procedures (gastrostomy [Q2510], vagotomy [Q2550], truncal vagotomy with gastrojejunostomy or pyloroplasty [Q2551], truncal vagotomy with gastrectomy [Q2552], gastroduodenostomy [Q2571], gastrojejunostomy [Q2572], or gastrojejunostomy, Roux-en-Y jejunostomy [Q2573]) and PPIs or H2-receptor antagonists (H2RAs) within 60 days of the procedure. Patients with malignant tumors (ICD-10 codes CXX.X) were excluded from our analysis to avoid false-positive results.

### Validation procedure

For the validation procedure, we obtained the medical records of 29 true PPU patients, ie, those who had been hospitalized in 2007 with PPU confirmed by upper endoscopy at either of 2 teaching hospitals in Korea—Ewha Womans University Hospital (EWUH) in Seoul and Seoul National University Bundang Hospital (SNUBH) in Gyeonggi Province. This cohort represented all true PPU patients that had been confirmed and hospitalized with PPU at EWUH or SNUBH in 2007; thus, no confirmed PPU patients at SNUBH and EWUH were left out of the analysis. This study was approved by the Institutional Review Boards of SNUBH and EWUH and by the internal auditing committee of the Health Insurance Review Assessment Service.

We identified possible PPU patients using the diagnostic modalities and Korean NHI claims database at SNUBH and EWUH in 2007, and those patients were compared with the true PPU cohort of 29 patients from SNUBH and EWUH in 2007. We assessed the predictability of our diagnostic algorithm by measuring PPV and sensitivity.

### Comorbidity

The Charlson Index was used as a summary measure to control for the influence of comorbid conditions on mortality after peptic ulcer perforation.^18^ This index has been validated for the use of hospital discharge data from ICD-based databases to predict long- and short-term mortality and was previously adapted for studies of peptic ulcer complications.^1^^,^^19^^,^^20^ Weights were assigned for 19 major disease categories, including peptic ulcer. Because ulcers were present in each patient, this condition was excluded from the calculation of the index. For all identified patients, the primary and secondary diagnoses of hospitalization before PPU events were included in the Charlson Index calculation. On the basis of definitions from previous studies, Charlson Index scores were classified as low (index score, 0), moderate (index score, 1–2), and high (index score, >2) to increase statistical power.^1^^,^^19^

### Other covariates

Patients were defined as “exposed” if they had been prescribed anti-ulcer drugs or ulcer-related medication within 30 days before the PPU event (ie, hospitalized with PPU). However, drug dose was not considered and over-the-counter NSAIDs could not be included in our analysis. Anti-ulcer drugs included PPIs (World Health Organization [WHO] Anatomical Therapeutic Chemical [ATC] classification system Code A02BC) and H2RAs (WHO ATC Code A02BA). Ulcer-related drugs included NSAIDs (based on WHO ATC Code M01A), aspirin, glucocorticoids (WHO ATC Code H02AB), and anticoagulants (warfarin and clopidogrel). With the exception of over-the-counter NSAIDs, all the included drugs are available only through prescription. A previous history of peptic ulcer hospitalization was defined as an event that occurred at least 1 year before the PPU event and had a primary or secondary diagnosis corresponding to ICD code K25.X K26.X, K27.X, or K28.X.

### Statistical analysis

The PPU incidence rate was calculated by dividing the number of patients with an initial PPU occurrence in 2006–2007 by the corresponding person-years in 2006–2007. Based on PPU hospitalization date, we constructed Kaplan–Meier survival curves and life-table estimates of 30-day mortality rates for the main study variables: age (<60, 60–79, ≥80 years), sex, and level of comorbidity (low, moderate, high). The associations between 30-day mortality and demographic characteristics were expressed as odds ratios, and significance was assessed using the χ^2^ test and Fisher’s exact test. Multivariate logistic regression with stepwise selection was conducted with a *P* value of 0.05 as the cut-off. All analyses were performed using SAS version 9.1 (SAS Institute Inc., Cary, NC, USA).

## RESULTS

### Validation of diagnostic algorithm

Our diagnostic algorithm and NHI claims database identified 26 potential PPU patients at Seoul National University Bundang Hospital and Ewha Womans University Hospital in 2007 (Table 1), 25 of whom were confirmed based on a chart review, yielding a PPV of 96% (25/26). One individual who had been identified as a PPU patient based on our algorithm—but was later found to be negative after the chart review—had secondary PPU (namely, iatrogenic PPU).

**Table 1.** Positive predictive value and sensitivity of diagnostic algorithm for perforated peptic ulcer

		PPU cases confirmed by chart review (gold standard)			
		
		Positive	Negative	Total	PPV
Possible PPU cases based on diagnostic algorithm and Korean NHI claims database	Positive	25	1	26	0.96
	Negative	4			
	Total	29			
	Sensitivity	0.86			

Abbreviations: NHI, National Health Insurance; PPU, perforated peptic ulcer; PPV, positive predictive value.

Medical records showed that 29 patients had been hospitalized with PPU at SNUBH or EWUH. Twenty-five of these individuals were matched with our possible PPU patients based on the diagnostic algorithm using the NHI claims database, indicating that the sensitivity of the algorithm was 86% (25/29). Out of 4 true PPU patients who were not captured by our algorithm, 2 did not have a primary or secondary diagnosis of K25-28 (their primary diagnoses were nontraumatic intestinal perforation and volvulus), 2 did not undergo predefined procedures (1 had acute posthemorrhagic anemia and the other had acute peritonitis), and 1 did not receive a PPI within 60 days of the procedure (acute peritonitis). Taken together, our results showed that correct ICD codes for PPU were not noted by doctors for 2 patients, and a PPI was not prescribed for 1 patient, which decreased the sensitivity of our algorithm.

### Incidence of peptic ulcer perforation

**Figure 2.**
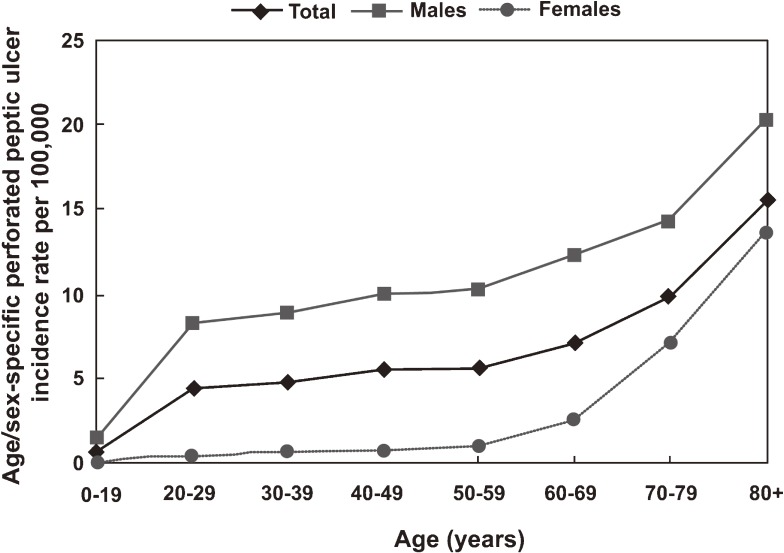
Annual age- and sex-specific incidence of perforated peptic ulcer per 100 000 Koreans in 2006–2007.

Descriptive statistics are shown in Tables 2 and 3. Overall PPU incidence in 2006–2007 was 4.4 per 100 000: 7.53 and 1.24 per 100 000 for men and women, respectively. Among the 4258 identified PPU patients, 73.8% were younger than 60 years, 85.7% were male, and 22.7% had at least 1 hospital discharge diagnosis that was included in the Charlson Index before the event date (Table 3). The predominance of males was greatest among younger patients, although this trend decreased with increasing age (Table 2). This is consistent with the data shown in Figure 2, which demonstrate that PPU incidence among women was less than 1 per 100 000 for those younger than 60 years. The rate was 0.98 per 100 000 for women aged 50 to 59 years, but it reached 10.28 per 100 000 for their man counterparts. The PPU incidence rate was consistently higher among men than among women, but this sex difference decreased among older patients (20.22 and 13.56 per 100 000 for men and women older than 80 years, respectively). Additionally, Table 2 shows that PPU patients older than 80 years were more likely to have some comorbidity (53.4%), and more likely to have been exposed to ulcer-related drugs (69.1%), as compared with younger individuals (<60 years).

**Table 2.** Demographic characteristics of Korean patients with perforated peptic ulcer (PPU) in 2006–2007

Variables	No. of PPU patients (%)		

	<60 years (*n* = 3143)	60–79 years (*n* = 892)	≥80 years (*n* = 223)
Sex			
Female	203 (6.5)	227 (31.0)	128 (57.4)
Male	2940 (93.5)	615 (69.0)	95 (42.6)
Comorbidity			
Low	2718 (86.5)	469 (52.6)	104 (46.6)
Moderate	353 (11.2)	303 (33.9)	91 (40.8)
High	72 (2.3)	120 (13.5)	28 (12.6)
PU-related hospitalization within 1 year			
No	3037 (96.6)	811 (90.9)	205 (91.9)
Yes	106 (3.4)	81 (9.1)	18 (8.1)
Ulcer-related drug^a^ use			
No	2277 (72.5)	364 (40.8)	69 (30.9)
Yes	866 (27.6)	528 (59.2)	154 (69.1)
Anti-ulcer drug use			
No	2625 (83.5)	767 (85.9)	210 (94.2)
Yes	518 (16.5)	125 (14.1)	13 (5.8)

**^a^**Ulcer-related drug is defined as NSAIDs (including aspirin and Cox-2 inhibitors), oral glucocorticoids, or anticoagulants (warfarin and clopidogrel).

**Table 3.** Univariate and multivariate analysis of risk factors and 30-day mortality rates for patients with perforated peptic ulcer

Risk factors	No. of patients (%)	No. of deaths	30-day mortality, %	Odds ratio (95% CI)	

				Univariate	Multivariate
Total	4258 (100)	135	3.17	—	—

Age (years)					
<60	3143 (73.8)	33	1.05	1.00	1.00
60–79	892 (20.9)	59	6.61	6.7 (4.31–10.30)^a^	2.76 (1.69–4.49)
≥80	223 (5.2)	43	19.28	22.5 (13.92–36.31)^b^	8.39 (4.81–14.1)^b^
Sex					
Male	3650 (85.7)	74	2.03	1.00	1.00
Female	608 (14.3)	61	10.03	5.20 (3.68–7.35)^b^	1.71 (1.14–2.56)^b^
Comorbidity					
0	3291 (77.3)	38	1.15	1.00	1.00
1–2	747 (17.5)	60	8.03	7.49 (4.99–11.22)^a^	3.85 (2.46–6.03)
3	220 (5.2)	37	16.82	16.44 (10.26–26.33)^b^	8.52 (5.1–14.31)^b^
PU-related hospitalization					
No	4053 (95.2)	118	2.91	1.00	Excluded^c^
Yes	205 (4.8)	17	8.29	3.07 (1.78–5.12)^b^	
Ulcer-related drug use					
No	2710 (63.6)	43	1.58	1.00	Excluded^c^
Yes	1548 (36.4)	92	5.94	3.92 (2.71–5.66)^b^	
Anti-ulcer drug use					
No	3602 (84.6)	134	3.72	1.00	Excluded^c^
Yes	656 (15.4)	1	0.15	3.92 (2.71–5.66)^b^	

^a^*P* < 0.05.

^b^*P* < 0.001.

^c^Excluded due to statistical insignificance on stepwise logistic regression, as indicated by *P* > 0.05.

### Thirty-day mortality rate

**Figure 3.**
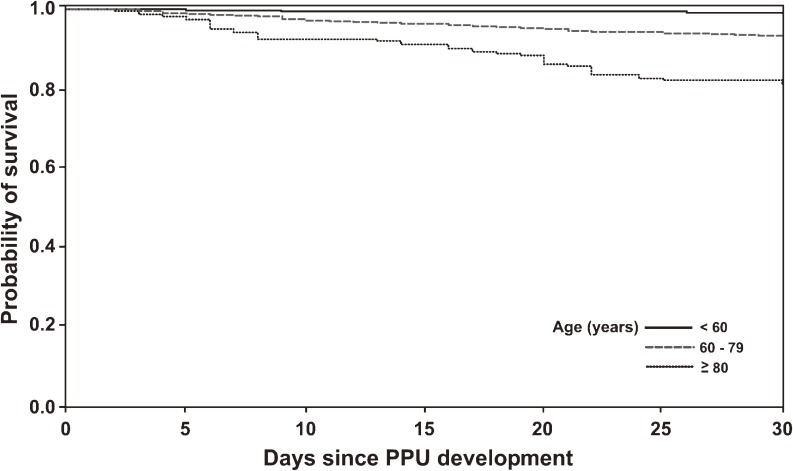
Kaplan–Meier plot of early mortality after perforated peptic ulcer development according to age. Early mortality was significantly higher in the oldest group than in the youngest group (log-rank test, *P* < 0.0001). The unadjusted odds ratios were 6.70 (95% CI 4.31–10.30, *P* < 0.001) and 22.5 (95% CI 13.92–36.31, *P* < 0.001) for patients aged 60 to 79 years and 80 years or older, respectively, as compared with patients younger than 60 years. PPU, perforated peptic ulcer.

**Figure 4.**
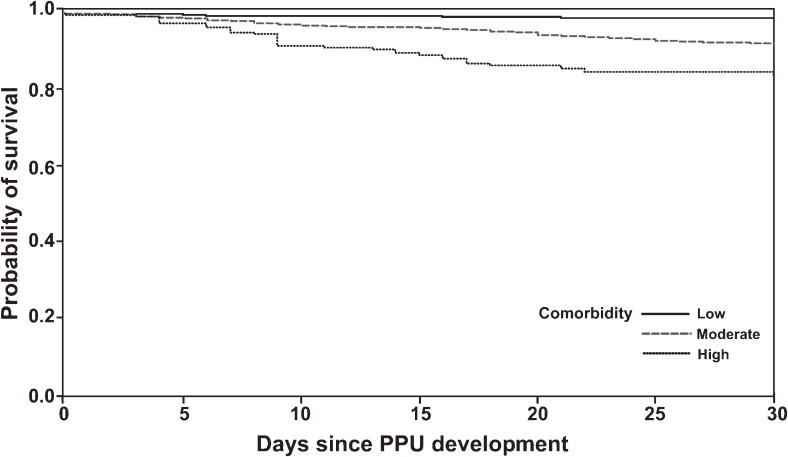
Kaplan–Meier plot of 30-day mortality after perforated peptic ulcer development, by level of comorbidity. Patients with high comorbidity had significantly higher short-term mortality as compared with patients with low comorbidity (log-rank test, *P* < 0.0001). The unadjusted odds ratios were 7.49 (95% CI 4.99–11.22, *P* < 0.001) and 16.44 (95% CI 10.26–26.33, *P* < 0.001), respectively. PPU, perforated peptic ulcer.

The overall 30-day PPU mortality rate was 3.17%, but this markedly varied with age, sex, and level of comorbidity. Table 3 shows that the 30-day mortality rate was 1.05% for patients younger than 60 years. The rate increased to 19.28% for patients older than 80 years (crude odds ratio 22.5, 95% CI 13.92–36.31, *P* < 0.001). Figures 3 and 4 show a consistent trend, suggesting that 30-day mortality significantly increased among patients aged 80 years or older, and among individuals with higher levels of comorbidity, as compared with patients younger than 60 years and those with low levels of comorbidity, respectively. In contrast to the PPU incidence rate, the 30-day mortality rate among PPU patients was higher for women (10.03%) than for men (2.03%; Table 3). Univariate analysis revealed that a prior history of PU hospitalization and previous ulcer-related and anti-ulcer drug exposure were also associated with short-term mortality. However, multivariate logistic regression identified age, sex, and comorbidity as independent predictors of mortality, and statistical significance disappeared for cases with a prior history of PU-related hospitalization and previous drug exposure. In particular, the mortality risk for patients older than 80 years was 8.39 times that of patients younger than 60 years. In addition, mortality was 70% higher among women than among men (Table 3).

## DISCUSSION

The incidence of peptic ulcer complications such as bleeding and perforation has decreased since *H. pylori* eradication became popular and the use of PPIs increased.^21^^–^^23^ As a result, the incidence of peptic ulcer peaked in 1910 and decreased gradually from the 1950s to the 1980s. Consequently, hospital admission, surgery, and death due to peptic ulcer markedly decreased.^24^ However, the results of a Korean multicenter study indicated that the prevalence of peptic ulcer was similar in 1995, 2000, and 2005.^25^ The incidence of gastric ulcer in tertiary hospitals also increased significantly between the early 1990s and 2006.^26^ Rapid increases in both the aged population and the use of ulcerogenic medications such as NSAIDs and aspirin may have caused these changes.^25^^–^^28^ Findings from these studies suggest that incidence and mortality due to peptic ulcer will increase.

Bleeding is the most common complication of peptic ulcer and is observed in about 15% of patients with peptic ulcer. The second most common ulcer-related complication is perforation, which reportedly occurs in as many as 6% to 7% of peptic ulcer patients.^29^ The development of pharmacologic and endoscopic approaches for treating peptic ulcer disease and its complications has substantially decreased the number of surgeries needed to correct this condition. Surgery is ultimately necessary for fewer than 5% of patients with peptic ulcer bleeding who require a transfusion and are unresponsive or refractory to endoscopic intervention.^29^ In contrast, most cases of PPU require surgery. Since the marked decrease in PPU incidence, especially after the 1990s, PPU data collection has become difficult even for a multicenter study conducted over several years. In our cohort study using the NHI claims database, the inclusion of a large number of PPU patients allowed us to estimate incidence and short-term mortality. To our knowledge, this is the first time such a large number of PPU patients have been analyzed.

The present study was limited by the fact that the data used for our analysis were from a national claims database rather than from hospitals. In addition, because validation was conducted based on 29 PPU patients from 2 Korean teaching hospitals (1 in Seoul and 1 in Gyeonggi province), the results might not be generalizable to the whole country. However, in a comparison of the demographic characteristics of confirmed PPU patients and the national cohort, the differences were not significant, eg, average age, 55 years (true cohort) vs 58 years (national); percentage of women, 10% (true cohort) vs 13% (national); Charlson Index higher than 2, 4% (true cohort) vs 5% (national) (data not shown). Also, since we included only hospitalized PPU patients, hospitals with an inpatient department (namely, general and teaching hospitals) rather than private clinics were likely to be the main venues of PPU treatment. For the purpose of validation, inclusion of smaller hospitals could have enhanced the generalizability of our results. However, since more than 90% of PPU patients present to tertiary hospitals (data not shown), we assume that our results are applicable to most PPU Korean patients. The Korean NHI covers most inpatient care services and strictly reviews claims submitted by health care providers. Thus, Korean physicians are especially attentive when they enter ICD and procedural codes. In addition, surgery is the main treatment method for PPU, although the condition can also be treated by hemoclipping and medical treatment with PPIs in carefully selected patients. Thus, treatment varies little between tertiary centers and general hospitals in terms of diagnosis and practice patterns, especially for hospitalized patients. Therefore, we believe that our results could be applied to the whole country. However, future research using data from a variety of medical centers is needed.

It might be argued that our selection of PPU codes is not PPU-specific, ie, that they could be used for other diseases, such as peptic ulcer bleeding or esophageal/colon ulcer perforation. Perforation due to other forms of ulcer is likely to have codes K25.X to K28.X as a primary or secondary diagnosis and to receive PPU-specific procedures; hence, such patients are not likely to be included unless they have other perforated ulcers in addition to PPUs. In our previous study of peptic ulcer bleeding (PUB) in Korea (Bae et al, *European Journal of Gastroenterology and Hepatology*, in press), we observed the types of medical procedures used to treat 115 confirmed PUB patients. Our predefined PPU-specific procedures were used for only 2 of 115 patients, both of whom had concurrent PPU and PUB. Thus, high PPVs coupled with the utilization pattern of true PUB patients suggest that our diagnostic algorithm results in a low number of false-positive patients.

Most PPU studies have been performed in European countries and included considerably older populations.^2^^,^^30^^,^^31^ The results of those studies indicated the importance of PPU risk factors such as advanced age and concomitant diseases. However, 1 study from the United Arab Emirates included PPU patients with a mean age of 35.3 years (range, 20–65 years) and identified different risk factors for perforation including smoking, history of peptic ulcer disease, use of NSAIDs, and daytime fasting.^32^ In the present study, we observed a sex difference in PPU incidence. This difference was stable for adults younger than 60 years but suddenly decreased among those aged 70 years or older. The greatest increase in PPU incidence was observed among men in their 20s. In addition, there was an increase in PPU incidence among elderly women, which has also been reported in a number of studies^31^^,^^33^^,^^34^ and might be due to the fact that older women are more frequently exposed to ulcer-related medications (such as NSAIDs) and are more likely have higher comorbidity—not because they experience delays in hospital admission. In contrast, peptic ulcer complications in relatively young men might be related to excess alcohol consumption and/or smoking.

We used 30-day mortality as an outcome measure for PPU patients. Because cause of death was not available, one might argue that it is hard to determine if death was caused by PPU or by other important extrinsic factors, such as delayed surgery/diagnosis or other comorbidity. However, in Korea, due to our extensive NHI coverage and easy access to care, delays in surgery are less of a concern, and patterns of clinical practice vary little in inpatient facilities. Thus, death within 1 month of PPU was more likely to be associated with PPU and comorbidity, which was captured by the Charlson Index. Therefore, although survival of PPU patients might not reflect PPU-specific outcome, it could be a proxy measure of short-term health outcome among PPU patients. Future research based on a nationwide collaboration might elucidate the causes of PPU.

In the present population-based study of more than 4000 PPU patients, the 30-day mortality among PPU patients was 3.17%, which was lower than in previous studies (4.2% to 31%).^2^^,^^7^^,^^8^^,^^35^^–^^37^ Our relatively low mortality rate could be related to the fact that the present study was performed using data collected from 2006–2007 in Korea, where medical facilities are easily accessible. However, like previous studies,^14^^,^^38^^–^^41^ we found that increased age, being female, and higher comorbidity were significantly associated with increased 30-day mortality. The higher mortality rate among women, which was also noted in previous studies,^2^^,^^42^^,^^43^ might be attributable to the fact that women are more likely to be exposed to over-the-counter medications (such as NSAIDs), or because the severity of PPU is actually higher among women than among men, which would not have been captured by our analysis. Future research should examine the factors associated with higher PPU mortality among women.

Exposure to ulcer-related drugs (such as NSAIDs), which was significant in univariate analysis, was not significant in multivariate analysis of 30-day mortality. Taha et al also reported that early mortality was associated with increased age and higher comorbidity but not with NSAID intake.^19^ This result could be due to the fact that older adults with comorbidities are more likely to take NSAIDs. Perhaps the effects of these drugs were obscured by age and comorbidities in multivariate analysis.

Our study has limitations. First, we utilized an administrative dataset; thus, information on clinical factors closely associated with PPU such as smoking, *H. pylori* infection, American Society of Anesthesiologists score, shock, and over-the-counter drug use could not be obtained. In addition, if diagnostic codes were not correctly recorded in the claims database, the Charlson Index could have underestimated comorbidity among PPU patients.

Identification of PPU cases based on ICD codes is especially controversial for the Korean NHI claims database, because use of a 4-character code (eg, K25.1 instead of K25) was optional until 2007. Thus, most entries in the Korean NHI claims database include only the first 3 digits of the ICD codes, which makes it impossible to use diagnostic codes in the Korean NHI claims database to differentiate peptic ulcer bleeding and peptic ulcer perforation from peptic ulcers without complications. This necessitates validation of PPU cases before performing studies using the Korean NHI claims database. The PPV of our diagnostic algorithm for PPU using the Korean NHI claims database was 96%, indicating that 96% of the PPU patients identified based on our algorithm were true PPU cases and only 4% were false-positives. The PPV of our study was much higher than those reported in previous validation studies,^11^^,^^12^ possibly because our diagnostic algorithm included procedural as well as diagnostic codes. In addition, our study demonstrated that an administrative database with proper case definitions can be a useful resource for PPU studies. Sensitivity in the present study was rather modest (86%). However, because higher sensitivity could compromise the PPV of the algorithm, we focused on achieving a higher PPV, due to the purpose of our study, ie, identifying true PPU patients using an administrative database.

In addition, we could not determine whether PPU was the reason for hospitalization or if PPUs developed during treatment for other diseases. However, if the primary diagnosis was PPU, then PPU was regarded as the primary disease affecting the patient. Like other administrative databases, the Korean NHI claims database has inherent limitations, including coding inaccuracy and incompleteness.^44^ However, since the importance of ICD codes is repeatedly emphasized to physicians who enter such codes in Korea, these errors have become less frequent.

In conclusion, our results showed that PPU incidence markedly increased among seniors. Age, female sex, and high comorbidity were strong predictors of poor health outcomes among Koreans with PPU. Special attention should be paid to elderly women with high comorbidity who develop PPU.

## ONLINE ONLY MATERIALS

eTable is available on the journal’s website at http://dx.doi.org/10.2188/jea.JE20120056.

eTables.
