# Supplementary material for: Incidence and Short-term Mortality From Perforated Peptic Ulcer in Korea: A Population-Based Study
Source: J Epidemiol. 2012 Nov 5;22(6):508–16. doi: 10.2188/jea.JE20120056 (PMC3798562; doi:10.2188/jea.JE20120056)
Supplement: eTables. [file je-22-508-s001.pdf]

eTable1. International Statistical Classification of Diseases and Related Health Problems, 10th revision (ICD-10) codes used to identify patients with potential peptic ulcer bleeding

ICD code	Description
**K25**	**Gastric ulcer**
K25.0	acute with hemorrhage
K25.1	acute with perforation
K25.2	acute with both hemorrhage and perforation
K25.3	acute without hemorrhage or perforation
K25.4	chronic or unspecified with hemorrhage
K25.5	chronic or unspecified with perforation
K25.6	chronic or unspecified with both hemorrhage and perforation
K25.7	chronic without hemorrhage or perforation
K25.9	unspecified as acute or chronic, without hemorrhage or perforation
**K26**	**Duodenal ulcer**
K26.0	acute with hemorrhage
K26.1	acute with perforation
K26.2	acute with both hemorrhage and perforation
K26.3	acute without hemorrhage or perforation
K26.4	chronic or unspecified with hemorrhage
K26.5	chronic or unspecified with perforation
K26.6	chronic or unspecified with both hemorrhage and perforation
K26.7	chronic without hemorrhage or perforation
K26.9	unspecified as acute or chronic, without hemorrhage or perforation
**K27**	**Peptic ulcer, site unspecified**
K27.0	acute with hemorrhage
K27.1	acute with perforation
K27.2	acute with both hemorrhage and perforation
K27.3	acute without hemorrhage or perforation
K27.4	chronic or unspecified with hemorrhage
K27.5	chronic or unspecified with perforation
K27.6	chronic or unspecified with both hemorrhage and perforation
K27.7	chronic without hemorrhage or perforation

K27.9	unspecified as acute or chronic, without hemorrhage or perforation
**K28**	**Gastrojejunal ulcer**
K28.0	acute with hemorrhage
K28.1	acute with perforation
K28.2	acute with both hemorrhage and perforation
K28.3	acute without hemorrhage or perforation
K28.4	chronic or unspecified with hemorrhage
K28.5	chronic or unspecified with perforation
K28.6	chronic or unspecified with both hemorrhage and perforation
K28.7	chronic without hemorrhage or perforation
K28.9	unspecified as acute or chronic, without hemorrhage or perforation
